# Enterocolitis, Cholera Infantum, Dysentery and Painful Dentition

**Published:** 1904-09

**Authors:** E. Van Goidtsnoven

**Affiliations:** Atlanta, Ga.


ATLANTA

### Journal-Record of Medicine.

Successor to Atlanta Medical and Surgical Journal, Established 1855, and Southern Medical Record, Established 1870.

Vol. VI. SEPTEMBER, 1904. No. 6.

BERNARD WOLFF, M.D., EDITOR, Nos. 319–20 Prudential.

M. B. HUTCHINS, M.D., BUSINESS MANAGER, 1007-1008 Century Bldg.

Published Monthly.

### ORIGINAL COMMUNICATIONS.

### ENTEROCOLITIS, CHOLERA INFANTUM, DYSENTERY AND PAINFUL DENTITION.*

By E. VAN GOIDTSNOVEN, M.D.,

Atlanta, Ga.

As the purpose of this paper is not intended to be an extended dissertation on the nature and pathology of the diseases respectively, I have, to avoid confusion and repetition, associated these four allied disorders. The three diseases, and usually, the last (if it be entitled to a distinctive designation as a disease), besides other points of similarity, have one symptom—increased frequency of the alvine dejections—common to them all. Authors of very high repute have sought to establish distinctions based upon peculiar characteristics of the stools and locality of morbid lesions; yet all admit that, clinically, it is impossible to determine with accuracy the pathognomonic differences. The local morbid lesions are to be found, more or less defined, in the gastrointestinal mucous

*Read before Atlanta Society of Medicine July 21, 1904.

membrane, most usually in the larger intestine. Enterocolitis and dysentery are nosological terms, intended to represent the precise anatomical locality of some grade of an inflammatory process, having its beginning, if not also its termination, in the mucous membrane of the digestive tube; yet it is not always easily determined where enterocolitis terminates and dysentery begins, or where cholera infantum ends and enterocolitis commences. The choleriform stools of cholera infantum may eventuate in the establishment of either enterocolitis or dysentery, and if frequent muco-sanguinolent stools accompanied with tenesmus, abdominal tenderness, and fever, be accepted as pathognomonic of dysentery, many cases running the course of enterocolitis, terminate with well-defined dysenteric symptoms. Even so eminent a pediatrica as West, of London, recognizes no other difference than the grade of the morbid process.

If the frequent and exhaustive serous and choleraic stools, attended with rapid waste and collapse, define and limit cholera infantum according to the systematic classification of diseases, where can be classed that common form of simple diarrhea not usually attended with fever or abdominal tenderness? Is not the distinction one of degree rather than of kind?

The term enterocolitis implies an inflammation of some portion of the intestinal mucous membrane. West says the anatomical changes are found "chiefly, though not exclusively, in the large intestine," and that the affection of the small intestine is "secondary and subsidiary" to colitis. J. Lewis Smith found in his 81 autopsies, with a single exception, "lesions indicating inflammation of the mucous membrane of the colon," and "in a large proportion of the cases, also, ileitis." Bochut discovered constant change in the large intestine, and maintains that the "mucous membrane of the small intestine is the only one of the constituent parts of this organ which participates in the changes of enterocolitis," and, furthermore, that the lesions which it presents are to be found "in nearly all the subjects." Meigs and Pepper "invariably found," in their numerous autopsies of cases of enterocolitis, inflammatory lesions of the large intestine, in some cases limited to it, in others extending to the small, and maintain that it is "clearly established that inflammation is much more frequently found in the large than in the small intestine. Notwithstanding these very decided opinions, based upon post-mortem investigations, experience and observation, affirmed by post-mortem examinations, demonstrate the uncertainty of the clinical distinction between inflammatory and non-inflammatory diarrhea. For, with all the symptoms of the former present during life, the autopsy may fail to disclose any inflammatory lesion appreciable to the naked eye. In 127 autopsies of children who had died of intestinal diseases, Rilliet and Barthez discovered the characteristic appearance of enterocolitis in 84 cases which had presented the symptoms of that disease; "in 24, though no symptoms had existed during life, similar changes were discovered; in 19 the signs of disease were present during life, but its morbid appearances were absent." West has enjoyed a similar experience and Bouchut and Smith have occasionally failed to discover any morbid lesion. M. Bertin in 57 autopsies failed in 4 to recognize any appreciable lesion. And M. Ballard expresses the opinion that children at the breast may have diarrhea without a trace of inflammation of the intestines. If these be facts they establish nothing more than that a certain array of symptoms represents, in one class of cases, certain well-marked morbid lesions, and in another class nothing. To seek explanation of the absence of any morbid lesion by ascribing the symptoms to any functional disorder is simply the admission that functional disturbance may eventuate in tissue-change, a fact well established. If hypersecretion, increased peristaltic action, and flux represent in one case functional disturbance and in another some grade of the inflammatory process, analogous reasoning would deduce the conclusion that the latter had its beginning in functional disturbance. If like causes under like conditions produce like symptoms, which in one class of cases terminated fatally without and in another class of cases with appreciable lesions, the logical inference would be that the difference was one of degree and not of kind. Functional activity augments secretion; so does simple hyperemia; and the two conditions may be correlative phenomena. In catarrhal intestinal inflammation it is perhaps impossible to determine, by the objective symptoms, which is the precedent condition. But the explanation of these discrepant results lies in the fact expressed in the *failure* of the investigator to find any morbid lesion. I am convinced that further and more accurate research will establish their identity in morbid process.

The simple diarrhea, so frequently supervening upon irregular, tedious and difficult dentition, ordinarily harmless in itself, and perhaps, salutary in the beginning, not unfrequently, in subjects favorably predisposed by a bad physical condition and improper hygienic surroundings, eventuates in the establishment of one of the more serious forms of intestinal diseases, and possesses, in this connection, no other significance than its causal relation to inflammatory and non-inflammatory diarrhea. Having its origin, as is almost universally held, in the development of the muciparous follicles of the intestinal mucous membrane, stimulated to excessive activity and increased secretion by the physiological process of dental evolution, during which period, with rare exceptions, a cholera infantum seizes its victims, selecting, most generally those of feeble, delicate and nervous constitution, the close relationship which the natural process bears to the disease as cause seems to be more than a mere coincident circumstance.

For convenience and comprehensiveness I have classified all the cases of non-inflammatory diarrhea under the head of enterocolitis. In doing this, I feel fully justified by the additional considerations that the latter is almost invariably preceded by the former, which, from neglect, injudicious or improper treatment, gradually and oftentimes inperceptibly develops the lesions characteristic of one of the most serious forms of disease. Rarely, if ever, does ileitis or colitis mark their inception. Hence, as West says, the distinction between them "is one of degree rather than of kind."

I am seeking to establish the identity rather than the unity of these diseases, and the foregoing considerations show the practical utility of these classical subdivisions and make apparent the danger of too much reliance upon the names rather than upon the symptoms. Undoubtedly, cases of these diseases occur, typical in their inception, symptoms, and progress; yet the far more frequent commingling of their phenomena renders classification difficult and, to a certain extent, valueless. The causes, symptoms and course, together with the age, physical condition, and hygienic surroundings of the patient, demand the attention and suggest the appropriate treatment.

Cholera infantum, so well marked by a group of symptoms, more distinctive in their general array than in their separate and pathognomomic significance and more formidable in their concurrent impressions upon the general system than in the special and solitary effect of any one of them, presents in typical cases the clinical features of a well-defined disease. The sudden onset of the assemblage of symptoms, their marvelous haste towards decisive and calamitous results, their terrible ravages, speedily expressed in the rapid prostration and exhaustion of the physical and vital powers, would seem to entitle this group to a distinctive appellation. Trousseau, in his graphic delineation of the disease, as it came under his observation, evidently had in view its similarity to Asiatic cholera, yet holding on to the doctrine, so constantly promulgated by him, and which Bouchut also maintains, that diseases owe their characteristic and distinguishing features to the specificity, he maintained that the points of dissimilarity are the "evident stamps of specificity." Its infrequency, as compared, with non-inflammatory diarrhea and enterocolitis; the occasional absence of appreciable morbid lesions, and, when present, their insignificance as compared to the gravity of the symptoms, and its frequent and direct association with dentition manifestly point to some constitutional peculiarity as an additional determining cause. This suggestion acquires force from the circumstance, believed to have been first suggested by Dr. J. F. Meigs, that in some families there exists a hereditary predisposition to the disease. More than once I have observed that the children of some families suffered from the graver disorder, while others in the immediate vicinity, and apparently living under less favorable hygienic conditions, escaped disease. The children of some families are never sick without becoming ill.

Dysentery, as nosologically defined, is so rare a disease during childhood, and especially during the earlier years, that some authors have denied its occurrence except as a secondary effect. Bouchut doubts its occurrence, and Vogel asserts that sporadic dysentery frequently occurs in infants. West, Smith, Meigs and Popper, Trousseau, Tanner and Ellis recognize the essential fact—the localized inflammation—but fail to indicate any other anatomical peculiarity of the morbid condition. In 514 cases of all diseases, I have memoranda of but 7 cases. These were sporadic. Basing my diagnosis upon the presence of fever, abdominal pain and tenderness, tenesmus, and muco-sanguinolent stools, I have felt compelled to separate them from the cases of enterocolitis, though the similarity of causes, conditions of life under which they appeared and the not unfrequent occurrence of cases of enterocolitis, in which the same symptoms ultimately appeared, strongly impress me with the futility of a nosological distinction. Epidemic dysentery is marked by peculiar phenomena; but as the disease ordinarily appears during infancy and during the summer months, the few cases being scattered here and there among a number of cases of enterocolitis and cholera infantum, all seemingly living under like conditions, the practical advantages of a distinct classification do not present themselves with much emphasis.

The great frequency of the diseases among the colored may, perhaps, find its explanation in the greater number of illegitimate births, and the consequent neglect and improper diet and feeding. Mothers without husbands and means of support are frequently compelled to obtain "service," among respectable and well-to-do families, to board their infants with those who pursue such business for a livelihood, only giving them the breast during a hurried visit morning and evening. During the intervals the child is frequently insufficiently and improperly fed. Numerous such instances have come under my observation, and Bouchut makes mention of the fact that in Paris non-inflammatory diarrhea is very prevalent among the infants of mothers who, "obliged to work in order to sustain their existence, quit their infants in the morning, return at several moments of the day to give them the breast." Apart from any consideration of the physiological or constituent alterations which may be induced in the mammary secretion, by the mental disquietude growing out of the conviction of moral turpitude or the privations of poverty, the "disagreement" of milk with the child, so frequently the alleged cause of the early weaning so generally practiced by these "at service mothers," may find its explanation in the watery condition of the milk, which has been shown by M. Peligot to be proportionate to the length of time it has been retained in the breast. Upon the consequences of this partial weaning and the subsequent improprieties of the feeding, I need not now dilate.

The influence of lactation, both natural and artificial, in the causation of infantile intestinal diseases, is far too frequently overlooked by the careless practitioner. Milk is the natural aliment of young animals, but the nursling is very frequently fed exclusively upon milk wholly deficient in the necessary nutrient and healthy constituents, and, indeed, often upon it when it is diseased. In this résumé of the causes I can not venture to discuss this subject in extenso, and might well content myself with a simple reference to the very full and instructive works of Routh on "Infant Feeding," and Donné on "Mothers and Infants," but I desire to impress the young practitioner with the importance and, in many instances, with the imperative necessity of searching for the cause in the habits, mode of life, and diet of the mother, and in the manner and frequency of nursing the sick infant. It is not only necessary to ascertain that the secretion is healthy in quality, but that it comes up to the proper standard in its normal constituents. The deleterious influence upon the lacteal secretion of sudden bursts of passion, of a nervous temperament, of menstruation and pregnancy, of excessive sexual indulgence, of irregular habits of life, and of certain articles of diet is too well established by clinical observation, if not by chemical analyses, to be considered as mere coincidences unworthy of the attention and investigation of the scientific physician. Dr. Decaisne has recently (*London Lancet, September, 1899*) shown that insufficient food may occasion very serious and varied disturbances of the quality of the milk. In his report to the Academie des Sciences of the results of his observations of forty-three women who nursed their infants during the siege of Paris, he deduced the conclusions that some women may, upon insufficient diet, produce abundant and rich milk and their children will thrive, while they themselves will emaciate; another class will produce but little milk and that very poor, and their children will suffer for want of nutriment and sicken with choleraic diarrhea, and a third class will produce scarcely any and their children will die. In syphilitic mothers the proportion of sugar is diminished and water increased in the milk; fever lessens and may suppress the secretion; emotion, mental anxiety and sorrow may diminish and also render it poisonous; puerperal fever seriously disturbs its healthy qualities; insufficient air, sedentary habits, and want of cleanliness not unfrequently impart to it conditions injurious to the health of the nursling. Certain drugs administered to the mother may affect the infant. Iodine can be detected in the milk; mercury given to syphilitic mothers will be conveyed to the child; opiates and some purgatives will demonstrate their physiological influence upon the sucking infant. All these circumstances and many others present considerations which demand the careful attention of the practitioner, and should not be overlooked in the study of the causes and in the application of remedies.

Without attempting to enumerate in detail the long list of causes so universally recognized as contributing to the production of these diseases, I will venture to suggest a few considerations: Whatever may be the influence of bad atmosphere and bad hygiene, something more than an elevated temperature, filthy streets, squalid lodgings and personal uncleanliness is necessary. Neither of the three diseases is peculiar to the children of the poor, or of the inhabitants of the narrow and foul alleys, or of the dwellers in the illy ventilated and stinking tenements of populous cities. They invade the palaces of the rich, the fashionable thoroughfares where dwell the families of leisure and affluence. Nor with all the care and vigilance of the health officer, nor with the lavish expenditure of the accumulated wealth of the millionaire can they be shut out from the nurseries of populous cities however cleanly, comfortable and luxurious the apartments may be. Nor are they exclusively confined to the illy and improperly fed, to the early weaned, the harshly treated, or the imprudently exposed, for all these causes are, presumably, of as frequent and constant occurrence during the winter as during the summer months; yet, it is during June, July, August and September that the wide-spread epidemics annually occur.

I have no doubt but many of my colleagues, who have listened more or less patiently to what has just been read, will pigeon-hole it to the cells of everlasting oblivion as so much that is obsolete and superannuated.

In science as in politics we should guard ourselves against conservative and innovating prejudices. Our contemporaries often mistake the novelty of an idea for a guarantee of its being entitled to the dignity of an uncontrovertible fact. The controlling aim and rule of our researches should be neither the old nor the new, *but the true.*

The contribution of my friend, Dr. H. F. Harris, to this most interesting topic will, do doubt, be greatly appreciated by the medical fraternity. I know of no one more competent to elucidate and to instruct on biological questions, and I will not presume to trespass on his fields.

Believing, as I do, in micro-organisms and autoinfection, I may be permitted to submit the following thoughts and suggestions:

Numerous theories have been offered to explain the action of bacteria in producing disease. The one which is accepted to-day is that the bacteria produce a chemical poison by splitting up pre-existing complex organic compounds in the body. These poisons are the ptomaines and from pure cultures of various bacilli, biologists have succeeded in isolating a particular ptomaine which when injected subcutaneously produce the symptoms of a disease invariably *sui generis.* Thus, for instance, Professor Vaughan of the Michigan University, isolated from milk and cheese a ptomaine which he named tyrotoxicon. From the investigations of Vaughan and the application of the knowledge thus obtained to his medical practice, Vaughan is of the belief that tyrotoxicon is the cause of many cases of enteritis and cholera infantum. As this poison appears very rapidly and abundantly in milk under favorable conditions, and furthermore, as the symptoms produced by tyrotoxicon and the pathological changes observed on post-mortem are identical with those occasioned by these diseases, this conclusion appears to be well-grounded.

In any case of gastro-enteric trouble, and I don't care what you may denominate it:

1. I prescribe *rest to the stomach.* All preceding food, natural or artificial, beef tea, cereals, broths, anything that requires and necessitates digestion is at once and peremptorily abolished. I order Panopepton and *that only* in their stead as it is *predigested.* This *regime* to be continued for several days until the character of the stools changes for the better. I also administer albumen water with a little table salt to promote osmosis and a few drops of whiskey to counteract shock and depression so frequent in these cases.

2. I relieve the portal circulation by sending an ultimatum to the germ, bacillus, bacterium, tyrotoxicon, whatever you may be pleased to call it; and this ultimatum is formulated as follows:

| | | |
|---|---|---|
| ℞ | Calomel | gr. i to ii |
| | Gray powder | gr. xvi to xxiv |
| | Sulphide of calcium | gr. ii to iv |
| | Sulpho carbolate of soda | gr. iii to xii |
| | Salol | gr. vi to xii |
| | | *M* |

Divide in chart xii.

S. One (1) powder every three (3) hours.

Also ℞ Wampole's Formolid: One tablespoonful to be diluted in a quart of water and injected once or twice a day.

3. In hydrocephaloid condition of the brain so frequent in cases of cholera infantum, I prescribe

| | | |
|---|---|---|
| ℞ | Bicoloride of mercury | gr. 1-20 |
| | Chloranodyne P. D. & Co | gtt. xx |
| | Chloroform water g. s | adf ℥vi |
| | | *M* |

S. One (1) teaspoonful every three (3) or four (4) hours.

4. In cases of muco-purulent, sanguinolent or dysenteric discharges, I first wash out the lower bowels with a warm saturated solution of boric acid to be followed immediately by an injection containing one-fourth of a grain of nitrate of silver diluted in half a pint of cold water, this to be done twice a day.

